# Milk and Milk-Contracts


**Published:** 1913-07-12

**Authors:** Everitt E. Norton

**Affiliations:** Medical Superintendent of Isleworth Infirmary.


### MILK AND MILK-CONTRACTS.*

By EVERITT E. NORTON, M.D., D.P.H., etc., Medical Superintendent of Isleworth Infirmary.

### VIII.—The Terms of a Milk-Contract (*continued*).

I suggest that the contractor should undertake that a special report in writing be sent to the purchaser, within twenty-four hours, of:—

(i) The occurrence in the case of anyone residing at, or working upon, any farm, or of anyone residing with such worker, of any notifiable infectious disease.

(ii) The visit to any farm of any medical practitioner, medical officer of health, veterinary surgeon, sanitary inspector, inspector of nuisances, inspector under the Food and Drugs Acts, or inspecting officer of a County Council, district authority, the Board of Agriculture, the Local Government Board, or other public authority. The report should give the name and address of the visitor, with the date and, if known, the purpose and result of the visit. The purchaser should, of course, grant permission in writing for the omission of any such report in the event, for instance, of repeated visits of a medical man attending in the case of a patient at a farm suffering from a non-infectious chronic illness. The visits of the purchaser's own inspectors should be reported.

(iii) Any failure, or known defect, in the ordinary water-supply or drainage of any farm.

(iv) Each instance in which any railway churn or can has been opened in transit, with the date and the name and address of the person by whom it was opened.

(v) The discovery that any cow is suffering from any diseased condition of the udder or from tuberculosis.

The purchaser should supply all necessary forms or books of forms, with counterfoils.

There are certain provisions which may, and which it is highly desirable should, be found in a milk-contract, but of which it is hopeless to demand the insertion under existing conditions. It is clear that the milk-trade must be left to take the initiative, realising that increased profits will accrue, in such matters as the delivery in London of chilled milk. I recommend that the purchaser should leave a space upon the tender-form and should state that special consideration will be given to any tender offering, even at an increased price, one or more of the following special advantages:—

(i) To supply milk cooled at the farm or farms to a defined low degree of temperature (say, 45° F.) and maintained, by methods to be approved, at or below that temperature until delivery.

(ii) To deliver the milk (cooled at the farms) in cooling churns, constructed to contain ice, or otherwise specially designed for the object in view.

(iii) To send the milk by rail in insulated or refrigerator vans used for milk-traffic only.

(iv) To deliver the milk within a stated time after milking, by road-motor conveyance or otherwise.

(v) To produce a certificate of a qualified veterinary surgeon that all the cows upon the farms from which it may be proposed to obtain milk have been recently tested by means of tuberculin, and have been proved by that test to be free from tubercular infection.

(vi) To produce a certificate of a qualified veterinary surgeon that all the cows at each of the farms from which it is proposed to obtain milk are healthy, and that the sanitary conditions and the methods employed are in each case in every respect satisfactory.

The purchaser should appoint a medical inspector and a veterinary inspector, and each should be authorised to represent and to make inspections, alone or with his colleague, on behalf of the purchaser.

The inspectors should be required, before the sealing of the contract, to advise the purchaser upon the details above mentioned and generally. They should visit and report upon every farm from which it is proposed to supply milk. They should advise concerning, and approve, the farm regulations, the strainers, the method and degree of cooling, the churns and their fasteners, and the details of any special offers made by the contractor in his tender, and should suggest any necessary modifications or additional safeguards. They should report, similarly, as required, upon any additional farm or farms proposed by the contractor for use as a source of supply. They should inspect and report from time to time, and when especially called upon, and should be responsible for seeing that the contract is being duly executed.

Both the medical and veterinary inspectors should deal with the cowsheds, their suitability and sanitary condition, including drainage, ventilation, and lighting, and their cleanliness; the cows, their housing and cleanliness, grooming, and washing; the cowshed methods, management, the avoidance of dust-raising work at milking times; the exclusion of children, dogs, and cats; the removal of manure and freedom from flies; the milk, its appearance and freedom from dirt, pus, etc.; the milkers, their cleanliness as to hands and clothes, the wearing of overalls, the forbidding of "wet-milking"; the supply of water, hand-basins, towels, etc.; the utensils, milking-pails, strainers, coolers, railway churns, etc., their patterns, conditions, and cleanliness, the manner and place of use and storage, the prevention of dust; the methods of washing and sterilising utensils, and the use of hot water, soda, and steam; the carriage of the milk, carts, railway vans, the avoidance of delay at railway stations and elsewhere, and of exposure of the churns to dust or sunshine.

* Previous articles appeared on May 3, 10, 17, 24, 31, June 14 and 28.

The medical inspector should also especially keep observation upon the water-supply, drainage, and general sanitary conditions of the farms, and the health of the workers.

The veterinary inspector should pay attention, in addition to the matters above mentioned, to the cows, their breed, age, health, and general condition; any cow showing signs of emaciation, tuberculosis, or any inflammatory or other disease of the udder; any cows added to the farm stock; cows having recently calved; and the food of the cows. Special attention should be directed by the inspectors to the condition of the cows and their cleanliness in winter, and of the utensils and their cleanliness in summer.

The purchaser should arrange with the medical inspector for the necessary occasional estimations of milk-fat, of solids not fat, of acidity, etc., and for such examinations as to bacterial content, volume of sediment, etc., as may be requisite.

In concluding this series of articles, I must repeat the words of Dr. Newsholme, which I have previously quoted: "Apart from the enforcement of public health regulations, public protection might be entirely secured under the ordinary conditions of commercial life if the public were willing to pay a little more for their milk and milk products." Dr. Newsholme referred to protection from tuberculosis, but his words are capable of a far wider application.

References.

A Report of an Investigation as to the Contamination of Milk, carried out on behalf of the Councils of Bradford, Hull, Leeds, etc., by Thomas Orr, M.D., B.Sc. (the "Yorkshire Joint Committee's Milk Report"), 1908.

Report to the Local Government Board on American Methods for the Control and Improvement of the Milk Supply, by Arthur Eastwood, M.A., M.D., 1909.

"The Nutrition of the Infant," by Ralph Vincent, M.D.

"The Milk Supply of Large Towns," *British Medical Journal,* 1903.

"The Pathology of Milk," by G. L. Eastes, M.B., B.Sc., *British Medical Journal,* 1899, Vol. II.

Report on the Milk Supply of Finsbury, by George Newman, M.D., D.P.H., F.R.S.E., 1903.

"Tuberculosis and the Milk Supply," by S. Delépine, M.B., M.Sc., *Lancet,* 1898, Vol. II.

"The Prevention of Tuberculosis," by Arthur Newsholme, M.D., F.R.C.P., 1908.

Milk and Dairies Bill; ordered by the House of Commons to be printed.

